# Augmented Harris Hawks Optimizer with Gradient-Based-Like Optimization: Inverse Design of All-Dielectric Meta-Gratings

**DOI:** 10.3390/biomimetics8020179

**Published:** 2023-04-24

**Authors:** Kofi Edee

**Affiliations:** Université Clermont Auvergne, Clermont Auvergne INP, CNRS, Institut Pascal, F-63000 Clermont-Ferrand, France; kofi.edee@uca.fr

**Keywords:** optimization, inverse design, metasurfaces

## Abstract

In this paper, we introduce a new hybrid optimization method for the inverse design of metasurfaces, which combines the original Harris hawks optimizer (HHO) with a gradient-based optimization method. The HHO is a population-based algorithm that mimics the hunting process of hawks tracking prey. The hunting strategy is divided into two phases: exploration and exploitation. However, the original HHO algorithm performs poorly in the exploitation phase and may get trapped and stagnate in a basin of local optima. To improve the algorithm, we propose pre-selecting better initial candidates obtained from a gradient-based-like (GBL) optimization method. The main drawback of the GBL optimization method is its strong dependence on initial conditions. However, like any gradient-based method, GBL has the advantage of broadly and efficiently spanning the design space at the cost of computation time. By leveraging the strengths of both methods, namely GBL optimization and HHO, we show that the proposed hybrid approach, denoted as GBL–HHO, is an optimal scenario for efficiently targeting a class of unseen good global optimal solutions. We apply the proposed method to design all-dielectric meta-gratings that deflect incident waves into a given transmission angle. The numerical results demonstrate that our scenario outperforms the original HHO.

## 1. Introduction

Harris hawks optimization (HHO) [1] is a metaheuristic optimization algorithm that provides a more intelligent and dynamic approach to search for optimal solutions compared to traditional optimization methods, such as gradient-based methods [2]. HHO is inspired by the cooperative hunting behaviour of Harris’s hawks and uses a population of hawks to search the solution space, with each hawk location with respect to the prey representing a potential solution. Compared to other metaheuristic optimization algorithms, such as genetic algorithm (GA) [3], particle swarm optimization (PSO) [4], ant colony optimization (ACO) [5], differential evolution (DE) [6], grey wolves optimizer (GWO), and whales optimizer (WO) [7,8], HHO offers a more cooperative approach for searching the solution space. The HHO hunting process uses two categories of hawks: exploratory hawks and exploitative hawks. Exploratory hawks are responsible for exploring the search space to find new areas with promising solutions, while exploitative hawks focus on exploiting the best solutions found so far. This division of labour allows HHO to balance exploration and exploitation, which can lead to better solutions compared to other metaheuristic optimization algorithms. HHO has shown promising results compared to other optimization methods in various studies. For instance, Senthilnath et al. [9] compared HHO to PSO, GA, and DE in solving multi-objective optimization problems in power system engineering. The results showed that HHO outperforms the other three algorithms in terms of its ability to find the Pareto-optimal front. Similarly, Baskar and Krishnakumar [10] compared HHO to three other optimization algorithms in solving an optimization problem in image processing. The results showed that HHO outperforms the other algorithms in terms of the accuracy of the solution obtained and the time taken to converge to the optimal solution. Moreover, HHO has been successfully applied to various other optimization problems. For example, Zhang and Cao [11] used HHO for multimodal optimization and its application in image segmentation. Sharma and Singh [12] proposed a hybrid approach for optimal power flow using HHO and an improved multi-verse optimizer. Mohajeri and Beheshti [13] presented a novel approach for feature selection using HHO. Lim and Lee [14] employed HHO for the optimal design of a shell-and-tube heat exchanger, while Qin and Yang [15] used HHO for joint base station placement and user association in heterogeneous networks. These studies demonstrate the versatility of HHO in solving various optimization problems in different fields, indicating its potential as an efficient optimization algorithm. Despite its advantages, HHO has some drawbacks. It is known to perform poorly in searching for optimal solutions and can have a deficiency in global search capability. Lately, the development of technology has led to further research in the field of intelligent algorithms, and various improvements have been proposed for the HHO algorithm to address these drawbacks. For instance, Kaveh et al. [16] proposed an efficient hybrid method based on the HHO and the imperialist competitive algorithm, compensating for HHO’s poor performance in searching for optimal solutions. Song et al. [17] identified HHO’s deficiency in global search capability and proposed the persistent-trigonometric-differences mechanism to improve its global search capability, while also enhancing the energy factor to better balance the algorithm’s exploration and exploitation.

We propose in this paper to improve the potential of the original HHO algorithm using useful feedback provided by a gradient-based-like method (GBL) [18]. The GBL optimizer employs the variations (“gradient”) of the figure of merit to iteratively update the potential solutions in the search space. In this method, a finite number of key design parameters are generated and iteratively updated during the process. In the case of meta-gratings made of nanorods, presented in this paper, the design parameters are associated with the widths of the nanorods and air gaps. These sequences of widths must simultaneously satisfy constraint equations. Due to these constraints, the design parameters are no longer linearly independent, and the notion of gradient becomes ambiguous. Therefore, unlike traditional gradient-based methods using adjoint method [19,20,21,22,23,24,25,26,27], that update all design parameters simultaneously, in the GBL approach, each variable is updated one by one accounting for the constraints involved in the design problem. In the strategy proposed in this paper, the GBL optimizer is used to target an initial population set through a minimal number of iterations in a short computational time. This pre-selected set of profiles is then used to initialize the Harris hawks optimizer. This hybrid strategy, denoted as GBL–HHO, is an optimal scenario allowing for efficient targeting of a class of previously unseen global optimal solutions. The proposed method is applied to design meta-gratings that deflect an incident wave into a given transmission angle. The polynomial modal method (PMM) [28,29,30,31,32] is used as an electromagnetic solver since it is more efficient, in the case of lamellar gratings, comparing to the Fourier modal method (FMM or RCWA) [33,34,35,36,37].

This paper is organized as follows: In Section 2, we briefly present the main principle of the GBL optimizer. In Section 3, we recall the key points of the classical Harris hawks optimizer (HHO). Section 4 is devoted to numerical results, where we compare the results obtained with the proposed hybrid GBL–HHO method to those of both the GBL method and the classical HHO. Additionally, to highlight the novelty of our approach, we successfully compare our results with other numerical optimization methods, such as the adjoint-based topology optimization (TO) method and global topology optimization networks (GLOnets) [38,39]. Through these comparisons, we demonstrate that the GBL–HHO algorithm significantly improves the potential of the original HHO by efficiently targeting a class of good global optimal solutions.

## 2. The Gradient-Based-Like (GBL) Optimization Method

**Figure 1:**
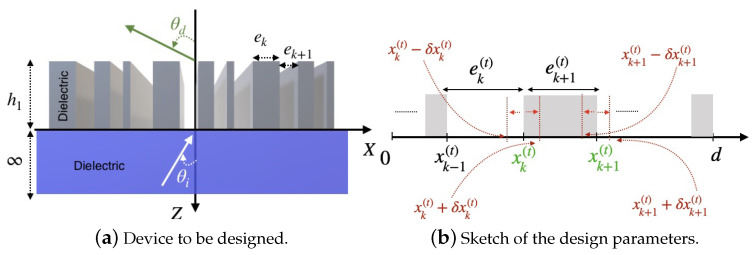
(**a**) Device to be designed. The device is made of dielectric nanorods of height $h_{1}$, backed on an infinite dielectric substrate. We consider a one-dimensional meta-grating consisting of Si nanorods with a refraction index of 3.6082, deposited on an SiO${}_{2}$ substrate (refractive index: 1.45). (**b**) Example of border-location variables $x_{k}$ update. Both widths and spacings of structures are optimized in order to increase the deflected efficiency into the desired diffracted order.

The first step in an optimization process is to define the objective that needs to be optimized and any constraints that must be satisfied. In our case, we aim to optimize a meta-gratings, composed of a finite number of nanorods Figure 1a, in order to improve the power deflected (transmitted) into a given diffracted order. The optimization process involves fine-tuning a sequence of key geometric parameters, including the size and relative positions of a given number of randomly distributed nanorods with random sizes. We may also impose minimum size constraints to achieve a high-performance meta-gratings design. Since both the nanorods widths and arrangement should be optimized simultaneously, both the nanorods and air-gaps widths, denoted by $e_{k}$, will be considered as the design parameters. See Figure 1b. The second step is to define the solution space, which involves specifying the range of values that the design variables $e_{k}$ in the objective function can take. The GBL optimization method can be viewed as a concatenation of three phases that are performed gradually and iteratively: Initialization, Evaluation of the fitness of the design parameters and Best current pattern update.

### 2.1. Initialization

The GBL algorithm starts by generating a finite number of key design parameters inside a given range. In the case of meta-gratings made of nanorods, these random variables are associated with both the nanorods and the air-gaps widths. The widths of the nanorods and air gaps are denoted by the design parameters $e_{k}$ which are calculated as follows:

(1) $$e_{k}=\left(e_{max}-e_{min}\right)r_{k}+e_{min}$$

Here, $r_{k}$ are a sequence of random variables in the range ${\left[0,1\right]}^{N_{p}}$, where $N_{p}$ is the number of design parameters, i.e., nanorod and air gap widths in the meta-gratings. These design parameters must satisfy simultaneous constraint equations, which are as follows:

(2) $$\left\{\begin{array}{c} \sum_{k=1}^{N_{p}}e_{k}=d\\ \;e_{k}\geq min\left(\mathrm{if}\;a\;\mathrm{minimum}\;\mathrm{size}\;\mathrm{feature}\;\mathrm{is}\;\mathrm{applied}\right) \end{array}\right.$$

Here, *d* is the size of the structure or its period in the case of meta-gratings. Since both the widths of the nanorods and air gaps must be updated while satisfying (2), it is suited to introduce a new sequence of variables called border-location variables, denoted by $x_{k}$. These variables are defined from $e_{k}$ as follows:

(3) $$x_{k}=x_{k-1}+e_{k},\;x_{0}=0,\;k\in \left[1,N_{p}-1\right]$$

The border-location variables, as shown in Figure 1b, enable easier fine-tuning of both the widths and locations of the rods within the optimization process.

### 2.2. Evaluation of the Fitness of the Design Parameters

At the *t*th iteration, a local variation $g^{\left(t\right)}\left(x_{i}\right)$ related to the objective function is computed at all nodes $x_{i}$ of the design area. The computation of these local variations involves induced fictitious currents when the system transitions from an old state to a new state, due to changes in its geometrical and/or physical parameters. Readers can refer to [40] for more details. In the design domain I, the discontinuous permittivity function ε(x) is described by a piece-wise constant function. The design domain I is divided into sub-intervals $I_{k}$, where each sub-interval is associated with a fitness $g_{k}^{\left(t\right)}$ defined as follows:

(4) $$g_{k}^{\left(t\right)}=\frac{\int_{I_{k}^{\left(t\right)}}xg^{\left(t\right)}\left(x\right)dx}{\int_{I_{k}^{\left(t\right)}}xdx}$$

where the fitness values $g_{k}^{\left(t\right)}$ are sorted in descending order at each iteration, and the location of each variable $x_{k}$ is searched to achieve an improvement in the objective. Due to the constraint Equation (2), the design variables $e_{k}$ are linearly dependent, meaning that they cannot be updated simultaneously, as is commonly performed in some gradient-based optimization algorithms that use adjoint methods [19,20,21,22,23,24,25,26,27]. As a result, the search for the optimal values of the design variables is performed iteratively. This approach ensures that the constraint Equation (2) is satisfied at each iteration while optimizing the objective function.

### 2.3. Best Current Pattern Update

In this phase, the fitness value $g_{k}^{\left(t\right)}$ is used to perturb the value of the variable $x_{k}$, taking into account the minimum size constraints. The perturbation can be either an ascending or descending increment, and only the increment direction leading to the best result is kept as the new optimal location of $x_{k}$. This new optimal location of $x_{k}$ is calculated as follows:

(5) $$x_{k}^{new}=best\left\{x_{k}^{\left(t\right)}-\delta x_{k}^{\left(t\right)},x_{k}^{\left(t\right)},x_{k}^{\left(t\right)}+\delta x_{k}^{\left(t\right)}\right\}$$

Here, $\delta x_{k}^{\left(t\right)}=\alpha^{\left(t\right)}g_{k}^{\left(t\right)}$ where $\alpha^{\left(t\right)}=a_{0}atanh\left(1-t/t_{max}\right)=a_{0}\beta^{\left(t\right)}$ is a parameter that decreases to zero with respect to the number of iterations. The constant $a_{0}$ is the rate at the first iteration, and $t_{max}$ denotes the maximum number of iterations. Once the best current sequence of variables $x_{k}^{new}$ is identified, a new sequence of the nanorod widths is computed using (6):

(6) $$e_{k}^{new}=x_{k}^{new}-x_{k-1}^{new},\;k\in \left[1,N_{p}\right],\;x_{0}=0,\;x_{N_{p}}=d$$

At this point, random oscillations are applied to the best current result, i.e., the vector $\left[e_{k}^{new}\right]$, via a random contraction or dilatation mechanism. The mathematical formalization of this random oscillatory behaviour is given by Equation (7):

(7) $$e_{k}^{new}+\beta_{0}\beta^{\left(t\right)}r_{k}$$

Here, $\left[r_{k}\right]=2rand\left(N_{p},1\right)-1$ is a vector of random variables in the interval ${\left[-1,1\right]}^{N_{p}}$, simulating an uncertainty of the oscillation mode of the current vector $\left[e_{k}^{new}\right]$. A parameter $\beta_{0}$ is added for fine-tuning the widths of the nanorods and air gaps, so that the induced perturbations do not change their values too much. In this paper, $\beta_{0}$ is set to $\beta_{0}=min_{k}\left(\left[e_{k}^{new}\right]\right)/\eta$. The parameter η is set to 5. Finally, Equation (8) is used to update the previous (old) geometry:

(8) $$e_{k}^{old}=e_{k}^{new}+\beta_{0}\beta^{\left(t\right)}r_{k}$$

**Figure 2:**
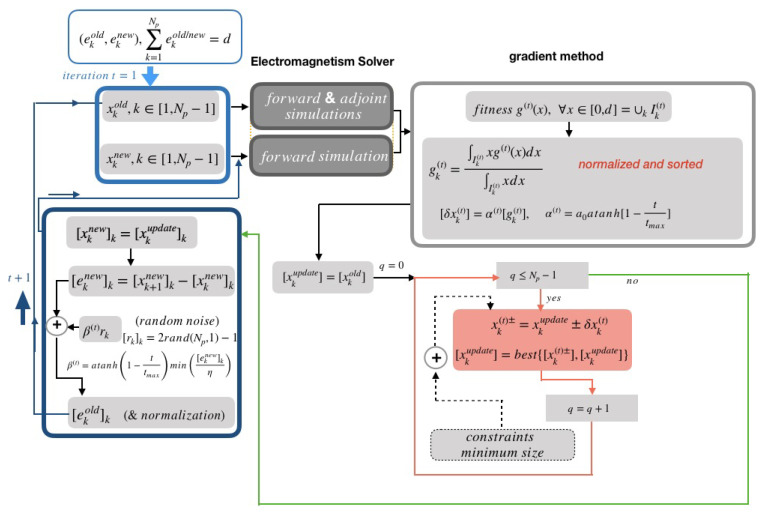
Flowchart of the proposed gradient-based-algorithm.

The whole process then restarts iteratively, gradually improving the figure of merit until it converges to a final structure. A flowchart of the proposed algorithm is shown in Figure 2.

## 3. Harris Hawks Optimizer

As reported in [1], the hunting process consists of two phases: exploration and exploitation. In the exploration phase, which is common in most hunting processes in nature, the hunter must wait until a prey is detected. Within the HHO framework, during this phase, Harris’s hawks search for prey by randomly perching at various locations. At iteration t+1, the location $X^{\left(t+1\right)}$ of a Harris hawk is updated according to the prey’s position in the previous iteration *t*, denoted as $X_{rabbit}^{\left(t\right)}$, or with respect to a randomly selected hawk’s location $X_{rand}^{\left(t\right)}$ in the whole population and the average location $X_{m}^{\left(t\right)}$ of the pack. This is the exploration phase. During the hunting process, while escaping the prey’s energy is assumed to decrease following the linear rule:

(9) $$E^{\left(t\right)}=2E_{0}\left(1-\frac{t}{T}\right)$$

where *T* denotes the maximum number of iterations and $E_{0}$, a random number belonging to [−1,1], represents the initial energy of the prey. Based on the escape energy value, hawks can decide to remain in the exploration phase by searching different landscapes, or to transit from exploration (if |E|∈[1,2]) to exploitation (if |E|<1) by searching for a local solution.

### 3.1. Exploration Phase

In this phase the prey has a high chance to escape since its energy *E* is high: |E|≥1. This phase is modelled by the following equation:

(10) $$X^{\left(t+1\right)}=\left\{\begin{array}{c} X_{rand}^{\left(t\right)}-r_{1}\left|X_{rand}^{\left(t\right)}-2r_{2}X^{\left(t\right)}\right|\;\mathrm{if}\;q\geq 0.5\\ \left(X_{rabbit}^{\left(t\right)}-X_{m}^{\left(t\right)}\right)-r_{3}\left(L_{B}+r_{4}\left(U_{B}-L_{B}\right)\right)\;\mathrm{if}\;q<0.5 \end{array}\right.$$

where $r_{i}$, (i=1,2,3,4) and *q* are random numbers belonging to [0,1] and

(11) $$X_{m}^{\left(t\right)}=\frac{1}{N}\sum_{i=1}^{N}X_{i}^{\left(t\right)}$$

with *N* being the total number of hawks.

### 3.2. Exploitation Phase (|E|<1)

When exploiting the neighbourhood, hawks can apply different strategies to capture the prey, depending on its remaining energy:

1. When |E|≥0.5, hawks consider the prey to still have enough energy to escape, and thus, a soft besiege strategy is applied. In this case, locations are updated as follows:
  (12) $$X^{\left(t+1\right)}=\Delta X^{\left(t\right)}-E\left|JX_{rabbit}^{\left(t\right)}-X^{\left(t\right)}\right|$$
  with $\Delta X^{\left(t\right)}=X_{rabbit}^{\left(t\right)}-X^{\left(t\right)}$, $r_{5}$, a random number in [0,1] and the parameter $J=2(1-r_{5})$ simulates the random jump strength of the prey during the escape procedure in each iteration. $X^{\left(t+1\right)}$ represents the updated location of the hawk at iteration t+1, $X_{rabbit}^{\left(t\right)}$ denotes the position of the prey at iteration *t*.
2. When |E|<0.5, hawks apply a more aggressive strategy, a hard besiege, to capture the prey, as they believe the prey to be too tired to escape. The location update equation for this phase can be written as:
  (13) $$X^{\left(t+1\right)}=X_{rabbit}^{\left(t\right)}-E\Delta X^{\left(t\right)}$$
  with $\Delta X^{\left(t\right)}$ always being the difference between the positions of the prey and the hawk.
3. In the exploitation phase, hawks can also perform some progressive rapid dives based on the Levy flight (LF) function. The LF function is defined as:
  (14) $$LF\left(x\right)=0.01\frac{u\sigma }{{\left|v\right|}^{1/\beta }}$$
  where *u*, *v* are random values within [0,1], and β is a default constant set to 1.5 in this paper. The σ term is calculated as:
  (15) $$\sigma ={\left(\frac{\Gamma \left(1+\beta \right)\times sin\left(\frac{\pi \beta }{2}\right)}{\Gamma \left(\frac{1+\beta }{2}\right)\times \beta \times 2^{(\beta -1)/2}}\right)}^{1/\beta }$$
  where Γ denotes the gamma function. Rapid dives can be performed with either soft or hard besieges. The hawks’ location at iteration (t+1) is evaluated based on the following equation:
  (16) $$X^{\left(t+1\right)}=\left\{\begin{array}{c} Y\;\mathrm{if}\;\;\mathrm{if}\;F\left(Y\right)<F\left(X^{\left(t\right)}\right)\\ \;Z\;\mathrm{if}\;\;\mathrm{if}\;F\left(Z\right)<F\left(X^{\left(t\right)}\right) \end{array}\right.$$
  - In the case of a soft besiege with progressive rapid dives
    (17) $$Y=X_{rabbit}^{\left(t\right)}-E\left|JX_{rabbit}^{\left(t\right)}-X^{\left(t\right)}\right|$$
  - In the case of a hard besiege with progressive rapid dives
    (18) $$Y=X_{rabbit}^{\left(t\right)}-E\left|JX_{rabbit}^{\left(t\right)}-X_{m}^{\left(t\right)}\right|$$
  in both cases Z=Y+S×LF(D), where *D* denotes the dimension of the problem and *S* is a random vector with size 1×D.

**Figure 3:**
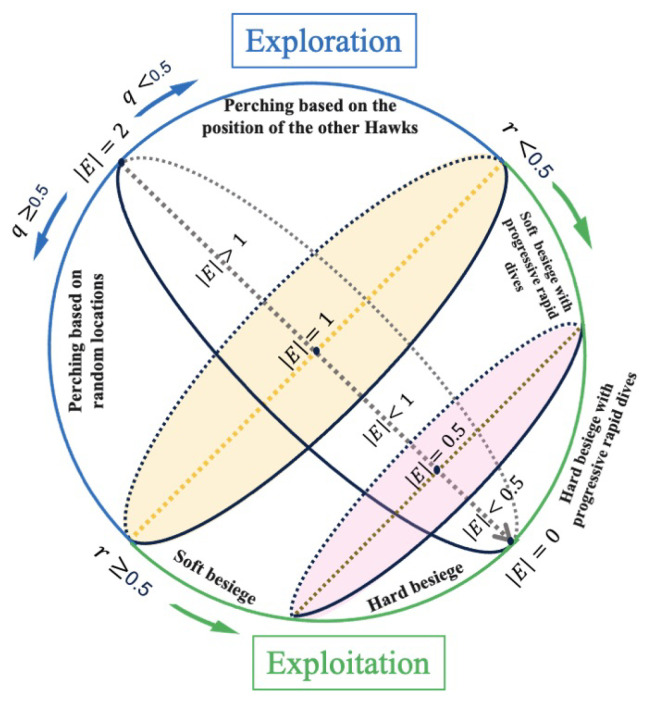
Sketch of the different phases of HHO.

The different phases of the HHO are summarized in Figure 3.

## 4. Results and Discussion

The efficiency of the concept proposed in this paper is demonstrated now through several numerical examples. We analyse the performance of the method in designing meta-gratings earlier studied in [38,39] that deflect a normally incident (incident angle $\theta_{inc}=0^{\circ }$) TM-polarized plane wave with a wavelength λ onto a given transmitted angle $\theta_{d}$ with the highest intensity. The calculation of the deflection efficiency for a given diffracted order of an incident plane wave is performed by dividing the electromagnetic field power travelling in the direction $\theta_{d}$, denoted as $P(\theta_{d})$, by the total incident power, denoted as $P_{inc}$. In other words, the deflection efficiency is equal to $P\left(\theta_{d}\right)/P_{inc}$. In all our studies, we consider a one-dimensional all-dielectric meta-grating consisting of Si nanorods with a refraction index of 3.6082, deposited on a SiO${}_{2}$ substrate (refractive index: 1.45). The grating’s height $h_{1}$ is set to 325 nm. The subtract is the incident medium, and the transmission region is the vacuum. To comply with standard fabrication techniques, a minimum size of both rods and air gap widths is set to $e_{min}$ = 50 nm within the optimization process. We investigate one wavelength, λ=0.9μm, and three deflection angles: small $\theta_{d}=40^{\circ }$, medium $\theta_{d}=60^{\circ }$ and large $\theta_{d}=80^{\circ }$. The parameters $e_{min}$ and $e_{max}$ of (1) are set to 50 nm and 100 nm, respectively.

**Figure 4:**
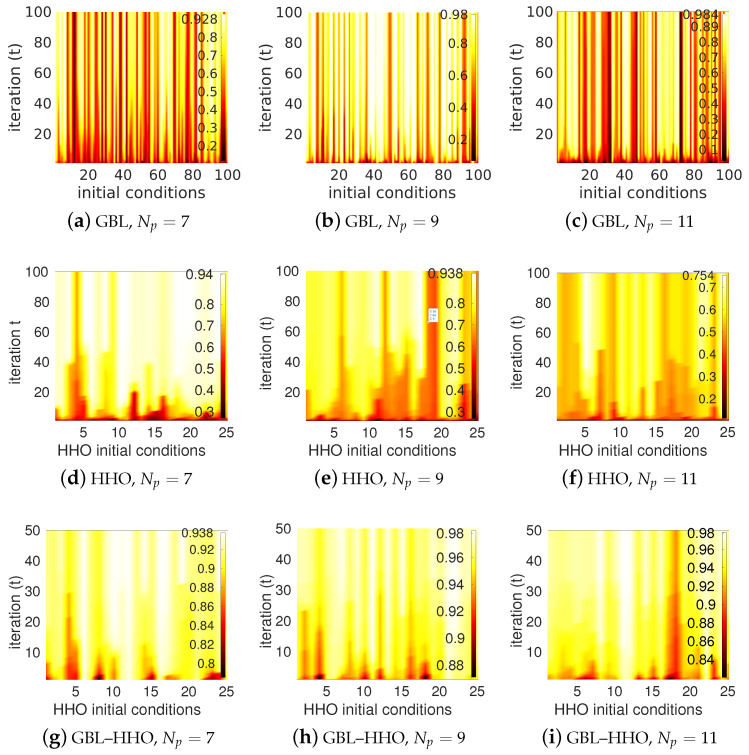
Convergence of the transmission efficiency into $\theta_{d}=60^{\circ }$ with respect to the number of iterations (t) (*Y*-axis) for different initial randomly generated profiles (*X*-axis), using the GBL, HHO, and GBL–HHO algorithms. Three values of the parameter $N_{p}$ are investigated: $N_{p}=7$ (3 nanorods + 4 air gaps), $N_{p}=9$ (4 nanorods + 5 air gaps), $N_{p}=11$ (5 nanorods + 6 air gaps). Numerical parameters: λ=0.9μm, deflection angle of $60^{\circ }$, polarization TM.

First, let us focus our analysis in detail considering the case of $\theta_{d}=60^{\circ }$. The GBL method is used to design the deflector. To perform the GBL method, one hundred sequences of $N_{p}$-tuple random variables $\left(\left[e_{k}^{old}\right],\left[e_{k}^{new}\right]\right)\in {\left[50\;\mathrm{nm},100\;\mathrm{nm}\right]}^{N_{p}}\times {\left[50\;\mathrm{nm},100\;\mathrm{nm}\right]}^{N_{p}}$ are initially generated, and for each pair of initial profiles, 100 iterations are performed in the optimization process. Three values of the parameter $N_{p}$ are investigated: in Figure 4a, $N_{p}=7$ (3 nanorods + 4 air gaps), in Figure 4b, $N_{p}=9$ (4 nanorods + 5 air gaps) and in Figure 4c, $N_{p}=11$ (5 nanorods + 6 air gaps). These figures show the convergence of the transmitted efficiency into $\theta_{d}=60^{\circ }$, $P\left(60^{\circ }\right)/P_{inc}$, with respect to the number of iterations (t) (*Y*-axis) for all 100 initial randomly generated profiles (*X*-axis). Results converge with respect to the number of iterations. However, the GBL method applied to initially randomly distributed nanorods yields locally optimized devices with highly variable efficiencies. This is consistent with the fact that the GLB optimization method is a local optimizer, and therefore it is very sensitive to the initial conditions. Basins of local minima appear in Figure 4a–c as red rays tearing the yellow background of high efficiencies.

Second, consider the classical HHO applied to our inverse design problem. In all the following examples, the minimum feature of the 1D device is still set to 50 nm, and *X* is the vector with components $e_{k}$ ($X={\left(e_{k}\right)}_{k\in [1:N_{p}]}$) simultaneously satisfying the constraints $\sum_{k=1}^{N_{p}}e_{k}=d$ and $e_{k}\geq 50$ nm. To perform the classical HHO, first, a set of *N* initial individuals, *X*-vectors, are generated. Second, HHO is performed within a given *T* number of iterations. Here, *T* is set to 100 and *N* is set to 50. As with all global optimizers performed on high-dimensional constrained problems, HHO could be sensitive to the initial conditions. That is why the algorithm is restarted with different randomly generated initial conditions. Here, HHO is restarted 25 times with different initial randomly generated sets of vectors *X*. Results are displayed in Figure 4d for $N_{p}=7$, Figure 4e for $N_{p}=9$, and Figure 4f for $N_{p}=11$. Let us analyse these results. From Figure 4d–f, one can observe, contrary to results of Figure 4a–c, that the results are less contrasted, as should be expected from a global optimizer. This indicates that the HHO is less sensitive to the initial conditions than the previous GBL method. However, the HHO algorithm is known to perform poorly in the exploitation phase, making the algorithm get prematurely trapped in a basin of local minimum. The higher the number of design parameters, the more likely the HHO will become trapped in local minimum pools. These phenomena are clearly illustrated in these figures by red streaks corresponding to low values of transmitted efficiencies. These red streaks increase with the number $N_{p}$ of design parameters since a higher number of design parameters results in a much larger search domain “dimension”. Therefore, the probability for the algorithm to be trapped in a basin of local minimum is lower in the case of $N_{p}=7$ than in the case of $N_{p}=9$, while $N_{p}=11$ is the worst case among these three examples.

Now, how can we combine both algorithms to efficiently solve the inverse problem under consideration? The proposed hybrid solution consists of two steps; in the first step, the GBL optimization method is applied to a set of initial geometries with a very few number of iterations ($T_{GBL}$). This is a pre-optimization phase. Typically, in this phase, ($T_{GBL}$) is set to 20, and only 50 initial geometries are randomly generated. After this $T_{GBL}$ small number of iterations, a new population of patterns is obtained. In terms of the requested objective, these individuals are slightly more efficient than the initial ones. In the second step, this new population is used for the initialization in the next HHO algorithm. HHO is then performed with a given *T* number of iterations. Numerical experience shows that T=50 is enough to achieve a stable solution. As in the case of the classical HHO, the whole process is restarted 25 times to check the sensitivity of the method to the initial conditions. The convergence of the results with respect to the number of iterations and for the 25 initial conditions is presented in Figure 4g–i. Images have low contrast compared to the GBL optimization method and classical HHO, indicating that the proposed hybrid method GBL–HHO is less sensitive to the initial conditions than the two previous optimization methods, namely GBL and HHO.

**Figure 5:**
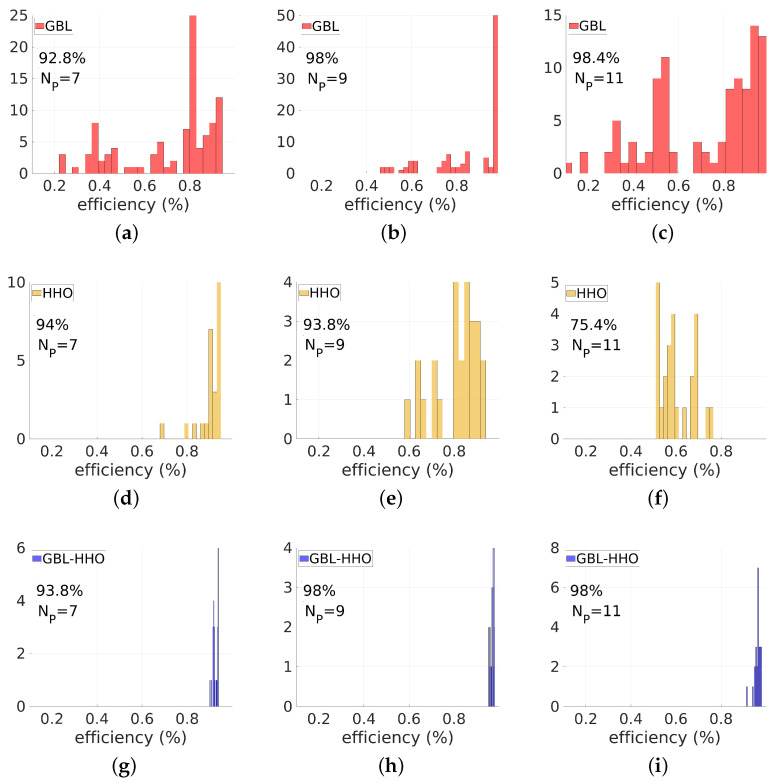
Comparison of performances of the GBL optimization (red), HHO (orange) and the proposed hybrid GBL–HHO algorithm (blue), for λ=0.9μm and the deflection angle of $60^{\circ }$. These figures display the efficiency histograms of devices designed using these three methods. Three values of the parameter $N_{p}$ are investigated: $N_{p}=7$ (3 nanorods + 4 air gaps), $N_{p}=9$ (4 nanorods + 5 air gaps), $N_{p}=11$ (5 nanorods + 6 air gaps). In (**a**–**c**), the GBL optimization is used. (**d**–**f**) are devoted to the HHO results. Results obtained from the proposed hybrid GBL–HHO are displayed in (**g**–**i**). The minimum size is set to $e_{min}=50$ nm.

Let us now compare the histograms of the transmission efficiencies and the highest efficiencies of the optimized devices obtained from these three methods. First, consider the histograms of efficiencies of the optimized devices shown in Figure 5 for the three methods and for different values of $N_{p}$. We still focus our investigation on the case of $\theta_{d}=60^{\circ }$. The histograms of efficiencies are narrower in the case of the HHO than in the case of the GBL optimization, regardless of the values of $N_{p}$. This is consistent with the fact that the GBL optimization method has the ability to reach an optimal solution with the cost of multiple runs by broadly spanning the design space. HHO can efficiently target a basin of good solutions but may be prematurely trapped in a local minimum pool in its exploitation mode. The histograms obtained from the GBL–HHO are the narrowest among all three methods. Even better, the GBL–HHO histograms are systematically narrow around high deflected power values, indicating that although the hybrid method could also be trapped in a pool of local optima, this pool is systematically a basin of the global optimum.

Let us confirm above observations by comparing the performance of highly optimized devices obtained by these three methods. These higher efficiencies are reported in Table 1 for the three methods, three values of $N_{p}\in \left\{7,9,11\right\}$, and for three values of $\theta_{d}$, $\theta_{d}=40^{\circ },60^{\circ },80^{\circ }$. However, continue to focus on a $60^{\circ }$-deflection angle. Regarding the results obtained using the GBL optimizer, high-performance solutions are reached regardless of the values of $N_{p}$. For $N_{p}=7$, the highest value of the transmitted power is 92.8% while the best four-nanorod device exhibits a transmission efficiency of 98% and a value of 98.4% is reached by the best five-nanorod device. When $N_{p}$ increases, the obtained optimal efficiency also increases, indicating that meta-gratings with a large number of nanorods, i.e., with small-width main features (narrow widths), should perform better than large-width ones. However, increasing $N_{p}$ also increases the design space, and the probability of reaching the basin of favourable solutions decreases. In other words, a significant part of the initial candidates could not yield these high transmission devices while increasing the number of nanorods. This is why the efficiency histograms of Figure 5 do not systematically narrow when $N_{p}$ increases, especially in the case of the GBL optimizer.

Contrary to the GBL method, the classical HHO is only efficient in targeting and reaching an optimal solution when the number of design parameters is low. For a high value of the number of design parameters $N_{p}$, HHO fails to reach the optimal solution in its exploitation phase. Using HHO, for $N_{p}=7$, the highest value of the transmitted power is 94%, which is higher than the GBL optimizer case (92.8%) but close to the case of the hybrid GBL–HHO (93.8%) method. When $N_{p}$ is increased to 9, the HHO performs poorly: the best four-nanorod device obtained by the HHO exhibits a transmission efficiency of 93.8%, which is less than both GBL optimization and GBL–HHO results (around 98%). For a higher value of $N_{p}$, namely $N_{p}=11$, the HHO is completely and prematurely trapped in a pool of local minimum: the maximum efficiency is no greater than 75.4% for the classical HHO, while satisfactory results of 98.4% and 98% are obtained from GBL optimization and GBL–HHO, respectively.

We performed a complementary analysis on devices operating with smaller and larger deflection angles: $\theta_{d}=40^{\circ }$ and $\theta_{d}=80^{\circ }$.

**Figure 6:**
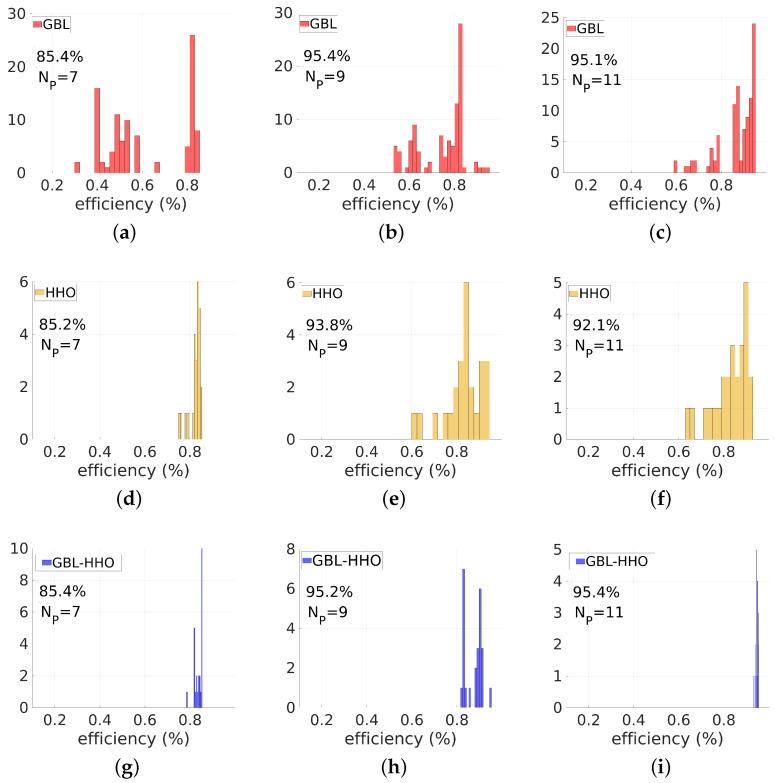
Comparison of performances of GBL optimization (red), HHO (orange) and the proposed hybrid GBL–HHO algorithm (blue), for λ=0.9μm and a deflection angle of $40^{\circ }$. These figures display the efficiency histograms of devices designed using these three methods. Three values of the parameter $N_{p}$ are investigated: $N_{p}=7$ (3 nanorods + 4 air gaps), $N_{p}=9$ (4 nanorods + 5 air gaps), $N_{p}=11$ (5 nanorods + 6 air gaps). In (**a**–**c**), the GBL optimization is used. (**d**–**f**) are devoted to the HHO results. Results obtained from the proposed hybrid GBL–HHO are displayed in (**g**–**i**). The minimum size is set to $e_{min}=50$ nm.

First, let us analyse the results of the small deflection angle case, i.e., $\theta_{d}=40^{\circ }$. Figure 6 shows the comparison between the efficiency histograms obtained with the GBL optimization, HHO, and GBL–HHO algorithms, for the three values of $N_{p}$. The highest transmission efficiencies obtained so far are also displayed in Table 1. The optimized devices at this shorter angle still have high performance. However, the performance of the optimized devices at $\theta_{d}=40^{\circ }$ tends to be lower than the $\theta_{d}=60^{\circ }$ grating. This lower performance may be explained by the fact that the grating period at $\theta_{d}=40^{\circ }$ is larger than the $\theta_{d}=60^{\circ }$-grating. We previously highlighted that meta-gratings perform better when their main features are small compared to the wavelength. Therefore, for a given wavelength, high-performance devices at smaller deflection angles may require a higher number of design parameters $N_{p}$.

**Figure 7:**
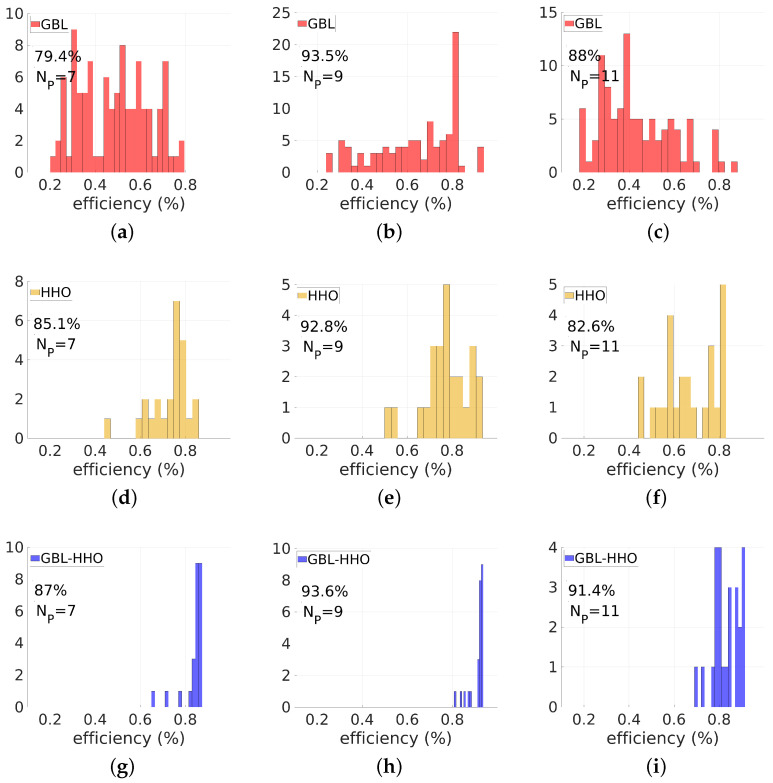
Comparison of performances of GBL optimization (red), HHO (orange) and the proposed hybrid GBL–HHO algorithm (blue), for λ=0.9μm and a deflection angle of $80^{\circ }$. These figures display the efficiency histograms of devices designed using these three methods and for three values of the parameter $N_{p}$: $N_{p}=7$ (3 nanorods + 4 air gaps), $N_{p}=9$ (4 nanorods + 5 air gaps), $N_{p}=11$ (5 nanorods + 6 air gaps). In (**a**–**c**), the GBL optimization is used. (**d**–**f**) are devoted to the HHO results. Results obtained from the proposed hybrid GBL–HHO are displayed in (**g**–**i**). The minimum size is set to $e_{min}=50$ nm. The highest deflected efficiency in each case is also displayed. For the numerical parameter, the efficiency distributions obtained from the GBL–HHO are narrower than those of GBL and classical HHO.

Let us consider the case of a large deflection angle. Generally, 1D meta-surfaces designed to deflect an incident plane wave onto large deflection angles have worse performance. In this case, it is difficult to exhibit high-performance structures from a set of random initial conditions, since the design space is more complex with a multitude of closely-spaced basins of local minima. Consequently, the GBL optimizer faces a multitude of basins of local minima and the classical HHO could also be trapped in these multitude of basins. Figure 7 shows the comparison between the efficiency histograms obtained with the GBL optimization, HHO, and GBL–HHO algorithms for optimized devices with λ=0.9μm and $\theta_{d}=80^{\circ }$. The third line of Table 1 shows the highest transmission efficiencies obtained for this deflected angle. Regarding the results of this table, GBL–HHO still systematically provides better results than the classical HHO at larger angle. This fact indicates that the algorithm definitively improves the possibility of the classical HHO to avoid trapping in undesirable local optimal solutions.

Numerical optimization methods, such as the adjoint-based topology optimization (TO) method and global topology optimization networks (GLOnets) [38,39], are increasingly utilized in photonics to optimize meta-surfaces performance. GLOnets, in particular, leverages machine learning techniques to efficiently solve complex optimization problems and is less reliant on the initial conditions. It is crucial to select the most suitable optimization method for a specific problem, and HHO algorithms and numerical optimization methods like the adjoint-based TO methods and GLOnets are all valuable tools for solving diverse optimization problems in photonics and beyond. The examples presented in the article, were previously introduced and studied in references [38,39]. Some results of these studies, reported on Table 2, demonstrate that GLOnets [38] outperforms other gradient-based TO methods for the selected example and in the current state-of-the-art techniques. Hence, the results obtained in this article can be compared with those obtained by GLOnets, as depicted in Figure 3 of reference [38], for the selected parameters, which include a wavelength of λ=0.9μm and diffracted angles of $\theta_{d}=40^{\circ },60^{\circ },80^{\circ }$. Comparing results of both tables, the proposed method, GBL–HHO, outperforms GLOnets in terms of the obtained highest diffracted efficiencies. Furthermore, the efficiency histograms obtained by GBL–HHO are narrower than those obtained by GLOnets, indicating that GBL–HHO is less dependent on the initial conditions than GLOnets.

**Figure 8:**
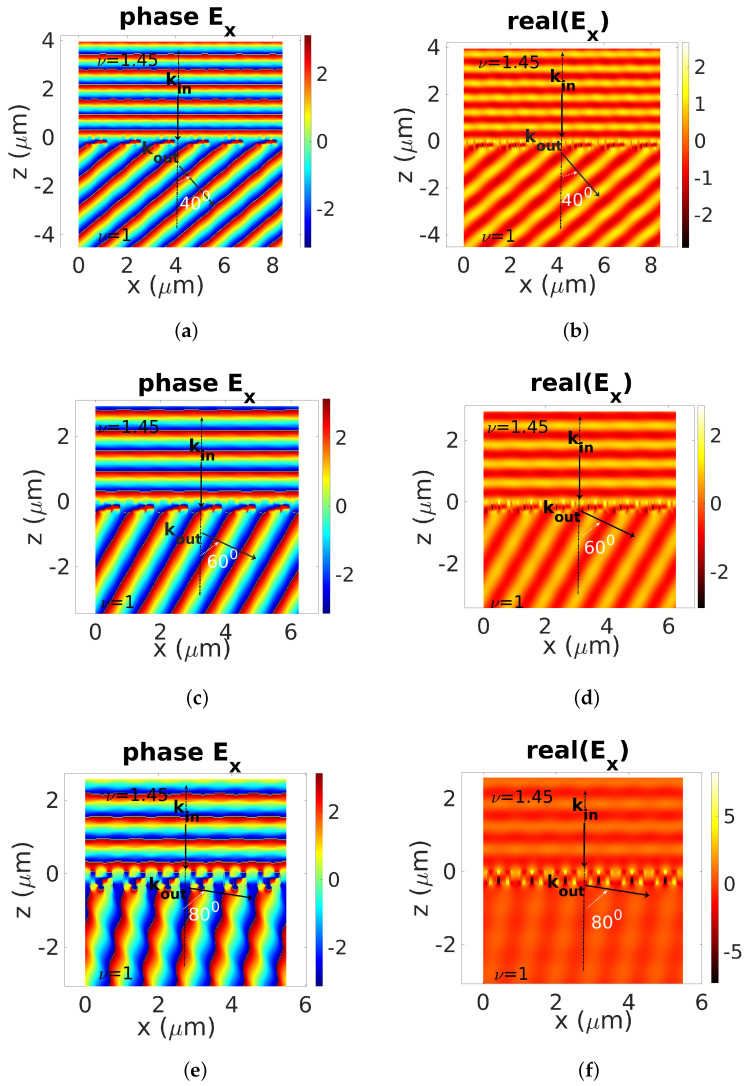
GBL–HHO applied to design a 1D high-transmission deflection meta-grating. Real part and phase of the electric field. Illustration of the quality of the deflection phenomenon supported by highest-transmission devices for three values of the $\theta_{d}$: $40^{\circ }$ (**a**,**b**), $60^{\circ }$ (**c**,**d**) and $80^{\circ }$ (**e**,**f**). Numerical parameters: λ=0.9μm, TM polarization.

The field patterns across the final highest-transmission devices are plotted in Figure 8. These figures display the phase and amplitude of the *x*-component of the electric field with respect to *x* and *z* for the previous three values of $\theta_{d}$: $40^{\circ }$ (Figure 8a,b), $60^{\circ }$ (Figure 8c,d), and $80^{\circ }$ (Figure 8e,f). With regard to the phases and real parts of the electric field, one can clearly distinguish the quality of the wavefront deflection phenomenon.

## 5. Conclusions and Outlook

In conclusion, the hybrid GBL–HHO optimization method leverages the strengths of both GBL and HHO techniques, providing a more efficient and effective solution for the inverse design of all-dielectric meta-gratings. This hybrid approach outperforms individual GBL and HHO methods, as well as other state-of-the-art methods, such as adjoint-based TO and GLOnets [38,39]. The GBL–HHO method successfully overcomes the limitations of the GBL optimizer, which is time-consuming and necessitates a large number of initial geometries to effectively cover the design space. Additionally, it addresses HHO’s propensity to become trapped in local optima, a situation that occurs when the algorithm prematurely transitions from the exploration to the exploitation phase. While reducing the number of iterations required for optimization, the hybrid GBL–HHO method increases the probability of finding a global optimum and streamlines the design process. It has demonstrated promising results in the inverse design of meta-gratings, laying the foundation for the development of innovative nanoscale devices with enhanced performance. Future research may concentrate on refining and extending the method, as well as investigating its potential application to other optimization problems across various domains. As the HHO algorithm has undergone numerous improvements, incorporating these advancements into the proposed hybrid method will also be a key focus in future work.

## Figures and Tables

**Table 1:** Panel of the efficiencies deflected onto diffracted orders of best devices using the three methods (GBL, HHO, and GBL–HHO) and for three values of $N_{p}\in \left\{7,9,11\right\}$. The best optimized device obtained from both the GBL optimizer and GBL–HHO have higher maximum efficiency values compared to those from the classical HHO. Numerical parameters: λ=0.9μm, $h_{1}=325$ nm.

Method		GBL			HHO		GBL–HHO		
$N_{p}$	**7**	**7**	**11**	**7**	**7**	**11**	**7**	**7**	**11**
$40^{\circ }$	85.4%	95.4%	95.1%	85.2%	93.8%	92.1%	85.4%	95.2%	95.6%
$60^{\circ }$	92.8%	98.0%	98.4%	94.0%	93.8%	75.4%	93.8%	98.0%	98.0%
$80^{\circ }$	79.4%	93.5%	88.0%	85.1%	92.8%	82.6%	87.0%	93.6%	91.4%

**Table 2:** Panel of the deflected efficiencies of best devices: comparison of adjoint-based TO and conditional GLOnet optimization. The results are obtained from Figure 3 of [38].

Method	Adjoint-Based TO	GLOnets Optimization
$40^{\circ }$	88%	87%
$60^{\circ }$	81%	94%
$80^{\circ }$	72%	89%

## Data Availability

Not applicable.
